# Simultaneous Analysis of 10 Priority PAHs in Iranian Sangak Bread Samples by Developing a GC-MS Method

**DOI:** 10.22037/ijpr.2020.113074.14097

**Published:** 2021

**Authors:** Farzad Peiravian, Attaollah Shakoori, Vahideh Moradi, Jamshid Salamzadeh, Arash Mahboubi

**Affiliations:** a *Department of Pharmacoeconomics and Pharma Management, School of Pharmacy, Shahid Beheshti University of Medical Sciences, Tehran, Iran. *; b *Vice-Chancellor for Food and Drugs Affairs, Shahid Beheshti University of Medical Sciences, Tehran, Iran. *; c *Food Safety Research Center, Shahid Beheshti University of Medical Sciences, Tehran, Iran. *; d *Department of Pharmaceutics, School of Pharmacy, Shahid Beheshti University of Medical Sciences, Tehran, Iran.*

**Keywords:** GC-MS, Poly Aromatic Hydrocarbons (PAHs), Sangak bread, Iran

## Abstract

Human exposure to polycyclic aromatic hydrocarbons (PAHs) is known as a carcinogen risk factor. In this study, a gas chromatography-mass spectrometry (GC-MS) technique combined with the QuEChERS extraction method was developed for concurrent analysis of 10 polycyclic aromatic hydrocarbons (PAHs) in Iranian traditional Sangak bread samples. The method was validated by determining different parameters, including; linearity, accuracy, precision, limit of detection (LOD) and limit of quantitation (LOQ). Calibration curves showed a linear relationship in the concentration range of 10-500 ng/g with a coefficient of determination (R^2^) ranged between 0.994 and 0.999. The obtained mean recoveries were 92-106% with the relative standard deviations (RSDs) in the range of 3-7% with an acceptable precision (RSD < 20%). The Limit of detections (LODs) for different PAHs were between 0.14-0.78 ng/g, while the limit of quantitation (LOQ) was 0.46-2.60 ng/g. Matrix effect studies showed that the analytes concluded signal suppressions or enhancements. Therefore, spiked calibration curves were used for overcoming this issue. The result of Sangak bread samples analysis using the validated method showed that 9 (19.4%) out of 47 Sangak bread samples were contaminated with phenanthrene (PHE) and anthracene (ANT) at the mean level of 10.08 ± 6.38 ng/g which were higher than the permissible limit of European Commission regulatory control value for BaP (1 μg/kg of wet weight) in processed cereal-based foods and baby foods for infants and young children.

## Introduction

Polycyclic aromatic hydrocarbons (PAHs) are semi-volatile, toxic environmental and food processing contaminants of pyrolysis, pyrogenic such as incomplete combustion of gas, wood, oil, coal or other substances and organic substances (1). They have particular concern because of their potential toxic, carcinogenic, and mutagenic properties. PAHs are significantly present in food as a result of heat processes such as smoking, grilling, toasting and drying and environmental contamination (2). Food is the main source of human exposure to PAHs throughout various routes such as air, soil or water (3,4). Cereal compounds, including bread are a major component of people’s food regimen. It is a good source of energy, contains vitamins, proteins, lipids, and minerals, which are essential in the human diet (5). For example, annually in the United States, Mexico, Finland, Poland, Germany, each person consumes cereal compounds of about 20, 31, 40, 59, 81 kg, respectively (6). In Iran, bread is also a major component of people’s food regimen and based on the national nutrition and food technology research institute of Iran, the average monthly consumption of bread is 117 kg per year which is very high in comparison with other countries (7). PAHs levels of bread depend on heating processes (time and temperature), the distance from the heat source, the amount of fat in the processed food and the kind of fuel besides the kind of process (8, 9 and 10).

Reports announced that amounts of PAHs in baked bread were 2–6 times higher than the initial flour and showed a variable distribution of these contaminants at parts of crust, loaf and crumb of the final product (4). The concentration raised from 1.07–3.65 ng/g detected in raw materials to 1.59–13.6 ng/g in bread baked at different temperatures (11). It is noticeable that major factors affecting in Bread’s contamination by PAHs are the contamination of bakery raw materials, primarily flour, and the baking process (9). In general, there are no regulations for PAH in bread, and according to the European Commission, the amount of benzo[a] pyrene (BaP) and PAH4 (sum of BaP, benz[a]anthracene (BaA), benzo[b]fluoranthene (BbF), and chrysene (CHR) in processed cereal-based foods and baby foods should not go beyond 1 ng/g (12). Rozentale *et al.*, showed the concentrations of PAHs in cereal products were in a range of 0.22-1.62 ng/g, with 14% of samples exceeding the current EU maximum permitted levels (5). 

The effect of toasting bread in the formation of PAHs has been studied, and the levels of PAHs in toasted bread were found in the range of 7.38–18.0 μg/kg (13). Some researchers also showed that the average PAH levels in baked bread by various fuels such as solar, solid waste, and electricity (14). 

Nowadays, different sample preparation methods have been set up for trace analysis of contaminants in various foodstuffs. QuEChERS, which stands for Quick, Easy, Cheap, Efficient, Rugged and Safe, was introduced by Anastassiades and collaborators for pesticide analysis (15). QuEChERS sample preparation, despite most analysis methods is time-saving and requires lower solvents. Also, the emerge of modern instrumental techniques like gas chromatography coupled mass spectrometer has greatly increased experts’ abilities to analyze various contaminants in complex matrices. 

Sangak is a favorite Iranian traditional bread. There are a few studies concerning the occurrence of PAHs in Iranian Sangak bread samples. Therefore, the essential aim of this research was the development and validation of a GC-MS method using QuEChERS sample preparation for the determination of 10 priority PAHs in traditional Sangak bread samples. Then, the validated method was applied to the analysis of PAHs in real Sangak samples.

## Experimental

*Chemicals and Analytical standards of PAHs*

The standard of 10 PAHs from the list of EU and EPA including; Acenaphthene (ACP), Fluorene (FLR), Phenanthrene (PHE), Anthracene (ANT), Fluoranthen (FLA), benzo[a]anthracene (B[a]A), chrysene (CHR), benzo[b]fluoranthene (B[b]F), benzo[k]fluoranthene (B[k]F), benzo[a]pyrene (B[a]P), and triphenyl phosphate (TPP) as internal standard were purchased from Sigma Aldrich /Fluka/Riedel-de-Haën (United States). All solvents including Ethyl acetate, acetonitrile and methanol (analytical grade for residue analysis, HPLC gradient grade) were purchased from Merck, anhydrous magnesium sulfate, sodium acetate and Bondesil-primary secondary amine (PSA, 40 μm) were supplied by Sigma Aldrich /Fluka/Riedel-de-Haën (United states). Deionized water was obtained from a Millipore Milli-Q water purification system.

*Standard Preparation*

All of the PAH compounds (Table 1) stock solutions were prepared by weighing exactly 10 mg of each standard, dissolved separately in 10 mL of ethyl acetate. A mixed intermediate standard solution at 5ng/g was prepared via the appropriate dilution of the stock solutions in acetonitrile (ACN). This solution was used as the spiking solution for the validation experiments. A stock solution of TPP in EtAc at a concentration of 20 ng/g was used as the internal standard, and an aliquot (50 μL) of this solution (20 μg/mL) was added to the spiked bread sample. All standard solutions were prepared in an amber color volumetric flask (to avoid light exposure) and stored at -20˚C whenever not in use. 

*Sample Preparation*

The QuEChERS method developed for the analysis of pesticides by Anastassiades et al. was used with some modifications to analyze PAHs in bread (15). Each sample was carefully ground and then, 5 g of each homogenized sample was accurately weighed and placed in a 50 mL centrifuge tube. Appropriate concentrations of the mixed working standard solution (for spiking) and 50 μL of the internal standard solution (200 ng/mL) were added to the tube, and 14 mLof ACN was added. The mixture was vortex mixed for 3.0 min, followed by adding a mixture of 2 g anhydrous MgSO4 and 1.5 g sodium acetate and subjected to vortex mixing for 3.0 min again. The mixture was centrifuged (Hettich, universal 320r from Germany) for 5 min at 9056 g, and 7 mL of the supernatant was then transferred into an appropriate tube placed in a nitrogen evaporator and dehydrated until dry. The residue was reconstituted in 0.5 mL ACN. The mixture was mixed for 3.0 min, followed by sonication for 3.0 min. Then the solution was transferred to a tube containing 60 mg anhydrous MgSO4 and 20 mg PSA. The mixture was vortex mixed vigorously for 1 min and centrifuged for 5 min at 18894 ×g. Finally, a 0.5 mL aliquot of the cleaned extract was transferred into an amber tube, and 1.0 μL was injected into GC-MS.

*GC-MS Analysis*

Gas chromatography (Model 7890 A, Agilent Technologies, USA) was employed using a mass spectrometry detector (Model 5975 C, Agilent technologies, USA) equipped with a split/splitless injector and an Agilent auto-sampler. A DB-5MS Agilent capillary column (30 m × 0.25 mm I.D., 0.25 μm film thicknesses) was used along with the following oven temperature program: initial temperature 80 °C, held for 2 min, 20 °C/min ramped to 140 °C, followed by 5 °C/min ramped to 315 °C. Helium (99.999%) was used as the carrier gas at a constant flow rate of 1 mL/min. The injection port, quadrupole mass analyzer, transfer line and ion source was adjusted at 300 °C, 100 °C, 280 °C, 230 °C, respectively, and the splitless mode was used. After acquiring the ion chromatogram in selected ion monitoring (SIM) mode, peaks were identified by their retention time and mass spectra. At least three ions were applied to the detection and determination of chemicals. The most abundant ion that had the highest signal-to-noise ratio and showed no evidence of chromatographic interference was selected for quantification. 

*Method validation*

Method efficiency is a substantial agent for all laboratories to guarantee the validity of the routine analysis. The method was tested to assess for matrix effect, linearity, limits of detection (LOD) and quantitation (LOQ), recovery and precision. Linearity was studied using the spiked calibrations method by analyzing six concentration levels using triplicate analysis over the concentration range of 10-500 ng/g. The spiked calibration curves (six points) for all the analytes were attained by plotting the ratio of peak area of each compound to that of internal standard against the concentration of the corresponding analyte. Recovery data were used to assess accuracy while their RSD was the day-to-day precision. To determine the mean recoveries and precision (repeatability) expressed as the coefficient percent of the variation, three spiked blank bread samples at concentration levels of 25, 50 and 200 ng/g were prepared and treated according to the procedure described in the sample preparation in triplicate. This was performed for three consecutive days. An aliquot of 50 μL of triphenylphosphate (TPP) solution in acetonitrile (20,000 ng/mL) was added to the spiked bread sample as an internal standard. The LOD and LOQ levels were calculated to evaluate PAH compound concentrations resulting in a signal-to-noise ratio of 3 and 10, respectively. Sample components impact on matrix effect by interfering gas chromatography and/or mass spectrometry (18). Bread sample, including salt, wheat protein and carbohydrates is no exception and can suppress or enhance targeted ions in mass spectrometry assay. Matrix effect (ME) was estimated by comparing the slopes of spiked calibration curves (A) and standard curves dissolved in extraction solvent (B) allowed the estimation of the recovery and matrix effect in terms of ion (19). The matrix effect was calculated as: 

ME (%) = (1 - A/B) × 100

*Determination of PAHs in Sangak samples*

Forty-seven traditional Sangak bread samples were provided based on the national standard of Iran (20) collected from Sangak bakeries located in Tehran city. Traditional bread was prepared by direct and indirect heating of natural gas at 170-300 °C. After collection, all samples were covered with aluminum foil and transported to the lab. Each sample was coded and dried to lose its moisture within one day. Then all of the samples were ground and stored in amber glass bottles at *−*20 *°*C until analysis. Finally, a 5.0 g portion of homogenized samples was weighted and analyzed. 

## Results and Discussion

*Determination of gas chromatography-mass spectrometry*

**Figure 1.**
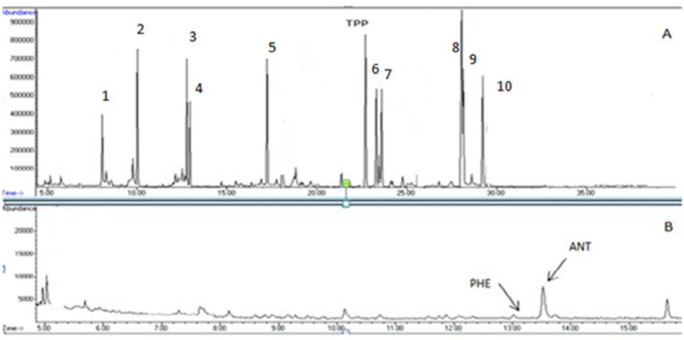
Chromatogram of the target PAHs in: (A) the standard mixture of 10 PAHs (TPP as the internal standard), (B) bread sample (Sangak). 1 (ACL), 2 (FLR), 3 (PHE), 4 (ANT), 5 (FLA), 6 (BaA), 7(CHR), 8(BbF), 9(BkF), 10 (BaP)

For analysis of the studied PAHs, optimization of GC and MS conditions was accurately conducted. Continuous injections obtained various GC parameters, including; MS conditions, oven temperature program, and define to a suitable column. Therefore, the SIM mode was employed for analysis of the studied chemicals. After acquiring mass data, one ion with higher intensity was employed as a quantification ion. For confirmation, at least two ions with the selected ion and their ion ratio and retention times were applied. Table 1 shows molar masses, SIM conditions, retention times, and related ion ratios for the studied PAHs. In addition, a chromatogram of the mixed standard related to 10 PAHs (A) also the same PAHs spiked in Sangak sample (B) was shown in Figure 1. 

*Method validation*

*Matrix effects*

Due to complexity, matrix effect (ME) is considered as the main challenge in food contaminant analysis. Matrix effect by signal enhancing or suppressing could affect the obtained results in analytical procedures. Hence it is necessary to examine this effect very well. There are various ways for studding the matrix effect. In this study, a solvent-based and spiked calibration curves approach was employed. Calibration standards are prepared by adding standard solution to blank bread samples subjected to the same sample preparation procedure. The calibration curve is constructed by analyzing in triplicate six concentration levels (10, 25, 50, 100, 200 and 500 ng/g). As shown in Table 2, the matrix effect for all of the analytes presents a strong signal enhance (>50%) except the matrix effect for BbF considered medium signal enhance whereas that was medium suppression signal in BaA. Therefore, spiked calibration curves were used to overcome the matrix effect, in this research.

*Linearity and linear range*

Spiked calibration standards at levels of 10, 25, 50, 100, 200 and 500 ng/g were prepared by the addition of 10, 25, 50, 100, 200 and 500 μL of standard stock solutions with a concentration of 5000 ng/mL to 5 g of blank bread samples in each case respectively. Quantification of the PAH compounds in bread samples was performed by using an internal standard method. Therefore 50 μL of TPP solution in acetonitrile (20,000 ng/mL) was added to all spiked bread samples. Calibration curves showed a linear relationship between the concentration and peak area ratios linear in the concentration range of 10-500 ng/g with a coefficient of determination (R^2^) ranging between 0.994 and 0.999. It shows that the extraction process and analytical method after validation have enough efficiency to determine PAHs at trace levels (Table 3). 

*LOD and LOQ measurement*

Limits of detection (LODs) and quantification (LOQs) were calculated based on the signal-to-noise ratio of equal to 3 and 10, respectively. The LODs and LOQs, as shown in Table 3, were between 0.139-0.785 ng/g and 0.459-2.591 ng/g, respectively.

*Recovery and precision*

The mean extraction recoveries were determined by applying the full procedure to triplicate samples in three consecutive days of analysis at three spiking levels, including 25, 50 and 200 ng/g with the same operator and laboratory. The percentage of mean recoveries obtained 92-106.0% for each PAH compound is considered an acceptable range of European Commissions, regulation (European Commi-ssions). Precision was expressed as relative standard deviation (RSDr) and evaluated like the recovery in different days. The average relative standard deviations (RSDs) of PAHs in bread was in the range of 3.16-7.78% with a satisfactory precision (RSDr < 20%) which were in the acceptable range of European guidelines (16, 17). the values of mean recovery and RSD percentage for each spiking level are presented in Table 4.

*Determination of PAH compounds in bread samples*

 As the results of the analysis of bread samples shown in Table 5, 47 Sangak samples were analyzed to evaluate the practicality of the method. The results showed that the PHE compound was detected in 6 samples (12.76%) at the mean level of 5.57 ± 5.21 which three samples were between LOD and LOQ and the 3 other samples were higher than LOQ. It is noticeable that all of the positive samples were detected in provided bread by undirected heating. On the other hand, 3 (6.38%) of ANT compound at the mean level of 14.59 ± 5.52 was detected in directed heating samples that all of them were higher than the amount of LOQ. 

Overall, 9 (19.4%) out of 47 Sangak bread samples were contaminated with PHE and ANT compounds at the mean level of 10.08 ± 6.38 ng/g which was higher than the permissible limit of European Commission regulatory control value for BaP (1 ng/g of wet weight) in processed cereal-based foods and baby foods for infants and young children (20). 

Previous studies have shown that there are few published papers in the field of PAHs in Iranian bread. Only research around the traditional bread (Sangak) was reported by Eslamizad *et al*. they developed a method for investigating BaP in Sangak bread (21). They analyzed 29 Sangak bread samples from Tehran’s bread bakeries in 2014. Results showed that two Sangak samples were contaminated with BaP. Their another study showed that 35.5% and 13% of Sangak bread samples collected in Tehran and Shiraz were contaminated with BaP respectively (22). In current study, besides of BaP, nine other compounds were analysed, but BaP was not detected in any samples. Research of Al-Rashdan et al on Seven Iranian bread samples showed NPH, FLR, and PHE were the most three abundant chemicals found in the studied breads (23). These findings were in accordance with Rashdan *et al.* studies. 

**Table 1:** Molar mass, quantification and confirmation ions in addition to ion ratio and retention times of investigated PAHs

**NO.**	**Compound**	**Abbreviation**	**Molar** **mass (g/mol)**	**Quantification Ions (m/z)**	**Confirmation Ions (m/z)**	**Ion Ratio**	**Retention Time (min)**
**1**	Acenaphtylene	ACL	152	152	152,151,76	4.509	8.18
**2**	Fluorene	FLR	166	166	166,165,82	1.176	10.16
**3**	Phenanthrene	PHE	178	178	178,176, 152	5.13	12.91
**4**	Anthracene	ANT	178	178	178,176, 152	8.441	13.09
**5**	Fluoranthene	FLA	202	202	202, 203, 201	6.76	17.40
**6**	Benz(a) anthracene	B(a)A	228	228	228.226,229	2.906	23.66
**7**	Chrysene	CHR	228	228	228.226,229	3.456	23.79
**8**	Benzo(b)fluoranthene	B(b)F	252	252	252, 253, 250	5.106	28.25
**9**	Benzo(k)fluoranthene	B(k)F	252	252	252, 253, 250	3.431	28.35
**10**	Benzo(a) pyrene	B(a)P	252	252	252, 253, 250	6.624	29.76

Note: Underlined ions represent selected confirmation ion.

**Table 2.** Matrix effects of studied PAHs

**NO.**	**Compound**	**Abbreviation**	**Spiked calibration curve**		**Solvent calibration curve**		**A**	**Matrix Effect (%)**
			**Slope**	**R** ^2^	**Slope**	**R** ^2^		
1	Acenaphtylene	ACL	0.002	0.998	0.023	0.997	0.098	90.15
2	Fluorene	FLR	0.005	0.997	0.020	0.999	0.249	75.12
3	Phenanthrene	PHE	0.005	0.998	0.010	0.994	0.490	50.94
4	Anthracene	ANT	0.003	0.998	0.008	0.999	0.410	58.98
5	Fluoranthene	FLA	0.007	0.999	0.018	0.994	0.379	62.13
6	Benz(a) anthracene	B(a)A	0.005	0.999	0.004	0.999	1.366	-36.64
7	Chrysene	CHR	0.006	0.999	0.013	0.998	0.423	57.65
8	Benzo(b)fluoranthene	B(b)F	0.008	0.999	0.012	0.999	0.705	29.52
9	Benzo(k)fluoranthene	B(k)F	0.003	0.999	0.009	0.999	0.341	65.91
10	Benzo(a) pyrene	B(a)P	0.006	0.992	0.016	0.995	0.393	60.71

**Table 3.** Linearity (R^2^), LODs and LOQs (ng/g) obtained for studied PAHs in Sangak samples

**No.**	**Compound**	**Regression Equation**	**Determination of Coefficient (R** ^2^ **)**	**LOD** ^a^	**LOQ** ^b^
1	Acenaphtylene	y = 0.002x + 0.038	0.998	0.651	2.150
2	Fluorene	y = 0.005x + 0.052	0.997	0.752	2.483
3	Phenanthrene	y = 0.005x + 0.077	0.998	0.611	2.015
4	Anthracene	y = 0.003x + 0.011	0.998	0.561	1.852
5	Fluoranthene	y = 0.006x - 0.006	0.999	0.139	0.459
6	Benz(a) anthracene	y = 0.005x + 0.012	0.999	0.243	0.803
7	Chrysene	y = 0.013x - 0.068	0.998	0.204	0.675
8	Benzo(b)fluoranthene	y = 0.008x + 0.015	0.999	0.349	1.152
9	Benzo(k)fluoranthene	y = 0.003x + 0.008	0.999	0.389	1.285
10	Benzo(a) pyrene	y = 0.017x - 0.106	0.994	0.785	2.591

^a^Limit of detection.

^b^Limit of quantification.

**Table 4.** Recovery (%) and repeatability (RSD_r_, %) studies of investigated PAHs in Sangak samples, spiked at 25, 50 and 200 ng/g levels (n = 9).

**No.**	**Compounds**	**25 (ng/g)**		**50 (ng/g)**		**200 (ng/g)**		**Average recovery ** **(n = 27)**	**Average** **RSDr** ** (n = 27)**
		**Recovery**	**RSDr**	**Recovery**	**RSDr**	**Recovery**	**RSDr**		
1	ACL	98.21	5.66	111.72	6.89	99.18	10.81	103.04	7.78
2	FLR	90.37	3.36	92.15	6.24	97.70	5.62	93.41	5.07
3	PHE	89.19	1.39	96.83	5.09	110.04	3.00	98.69	3.16
4	ANT	93.96	1.37	98.26	3.78	111.89	5.98	101.37	3.71
5	FLA	108.52	1.11	107.89	2.31	102.26	8.22	106.22	3.88
6	B(a)A	86.75	3.58	94.43	2.30	98.42	8.89	93.19	4.92
7	CHR	100.14	1.67	99.95	1.89	101.79	6.97	100.62	3.51
8	B(b)F	93.35	1.93	104.36	2.74	101.38	5.93	99.69	3.53
9	B(k)F	90.31	2.44	98.06	2.56	109.42	12.63	99.27	5.87
10	B(a)P	90.28	3.09	94.18	4.40	93.79	5.62	92.75	4.36

**Table 5:** PAHs values determined in Sangak bread samples (n = 47).

**No.**	**Compounds**	**No. of positive samples**	**Mean ± SD** **(ng/g)**	**Min Level** **(ng/g)**	**Max Level** **(ng/g)**	**No. of samples in the range ng/g**	
						**LOQ-LOQ**	**> LOQ**
1	ACL	0	0	nd	nd	0	0
2	FLR	0	0	nd	nd	0	0
3	PHE	6 (12.76%)	5.57 ± 5.21	0.64	11.06	3	3
4	ANT	3(6.38%)	14.59 ± 5.52	10.12	20.77	0	3
5	FLA	0	0	nd	nd	0	0
6	B(a)A	0	0	nd	nd	0	0
7	CHR	0	0	nd	nd	0	0
8	B(b)F	0	0	nd	nd	0	0
9	B(k)F	0	0	nd	nd	0	0
10	B(a)P	0	0	nd	nd	0	0
	∑10 PAHs	9 (19.14%)	10.08 ± 6.38	0.64	20.77	3	6

*nd: not detected.

## Conclusion

In the present study, the QuEChERS procedure coupled with gas chromatography-mass spectrometry was used to extract and determine ten polycyclic aromatic hydrocarbons in bread samples. This method is advantageous in terms of total solvent utilization and also the extraction solvent is less toxic. After optimization of the parameters affecting the analysis efficiency, the identification and quantification of the PAHs were carried out by applying the mentioned techniques for the analysis of bread samples. The results showed that applied method results in a satisfactory correlation coefficient, recovery and precision. Moreover, the use of spiked calibration curves for constructing the calibration curve substantially reduced adverse matrix-related effects. The results reveal that only 2 compounds of 10 analyzed PAHs were detected in traditional bread (Sangak) at the level of higher than maximum levels set for processed cereal-based foods and baby foods.
